# Prevalence and awareness of mode of transmission of typhoid fever in patients diagnosed with *Salmonella typhi* and *paratyphi* infections at the Saint Elisabeth General Hospital Shisong, Bui Division, Cameroon

**DOI:** 10.11604/pamj.2021.40.83.16893

**Published:** 2021-10-07

**Authors:** Heasla Fibuonu Njoya, Mbunka Muhamed Awolu, Tume Bonglavnyuy Christopher, Jutcha Florent Duclerc, Jerome Ateudjieu, Frankline Sevidzem Wirsiy, Catherine Atuhaire, Samuel Nambile Cumber

**Affiliations:** 1 Department of Biomedical Sciences PO Box 067, University of Dschang, Dschang, Cameroon,; 2 Pfizer Scholar, One Young World (OYW), 10 Queen Street Place, London, United Kingdom,; 3 Department of Nursing, Faculty of Medicine, Mbarara University of Science and Technology, Uganda,; 4 Office of the Dean, Faculty of Health Sciences, University of the Free State, Bloemfontein, South Africa,; 5 Institute of Health and Caring Sciences, Sahlgrenska Academy at the University of Gothenburg, Gothenburg, Sweden

**Keywords:** Typhoid fever, *Salmonella typhi*, prevalence, stool culture, Cameroon

## Abstract

**Introduction:**

typhoid fever is a systemic infectious disease caused by the bacteria Salmonella enterica subspecies (typhi). It is a major cause of morbidity and mortality worldwide. This cross-sectional descriptive study aimed at determining the prevalence and awareness of the mode of transmission of Salmonella typhi among patients at the Saint Elisabeth General Hospital Shisong of Cameroon.

**Methods:**

the study carried out from March 1^st^, 2017 to May 31^st^, 2017 recruited patients who presented at the hospital with clinical signs and symptoms of typhoid fever and who had lab requests for stool culture requested by the resident physician. The prevalence of Salmonella typhi infections among the patients and the proportion of patients with adequate knowledge on the mode of transmission of Salmonella typhi were estimated at a 95% CI. Data were analyzed using Epi info7.1.3.3.

**Results:**

out of the 172 patients recruited for the studies, 52 (30.1%) were diagnosed with Salmonella typhi, 59.6% of which were male. Also, 3 (5.8%) were diagnosed with Salmonella paratyphoid A. A positive correlation between knowledge on the mode of transmission of Salmonella typhi and the level of education was established, showing that 92% of participants with a higher level of education indicating that typhoid fever can be contracted through consumption of contaminated water.

**Conclusion:**

high prevalence of typhoid fever was observed in our study. The unawareness of the patients on typhoid fever and its contraction through contaminated water and food was positively correlated to the level of educations of the patients. These findings, therefore, suggest a public health challenge faced by inhabitants in this region where typhoid fever remains endemic. Scarcity of potable water, improper drainage systems, and problems of unsanitary toilets in Cameroon require urgent intervention.

## Introduction

Typhoid fever is a systemic infectious disease caused by the bacteria *Salmonella enterica* subspecies *(typhi)* and is the major cause of morbidity and mortality worldwide [1]. Enterica serotype *Typhi* is the etiological agent of typhoid fever. The illness begins with mounting fever, headache, vague, abdominal pain and constipation/diarrhea which may be followed by the appearance of rashes and a state of prolonged apathy, toxemia, delirium, disorientation, or coma, followed by diarrhea which if left untreated can lead to complications affecting various organ of the body [2].

In 2004 it was estimated that there were 21.6 million cases of enteric fever causing 200,000 deaths worldwide [3]. High prevalence was seen in areas with rapid population growth, increased urbanization, and limited safe water, infrastructure, and health systems like parts of India, South and Central America, and Africa. In such areas, typhoid is predominantly a disease of children where the main source of infection is stool excretion of *Salmonella typhi* during and after infection [4]. The incidence rate of typhoid in the Asia-pacific region is estimated to be more than 100 cases per 100,000 persons per year [4]. According to Gizachew *et al*. [5] this Infection occurs in all age groups but with higher incidence and more variable clinical presentation in children.

In a study by Mohammad *et al*. [6] diagnosis of typhoid fever in Dhaka using the Widal test and bacteriological culture showed that the prevalence of infection was higher among men than among women. In a study in Nigeria using the Widal test, Smith *et al*. [7] found the prevalence of typhoid fever to be higher among females. However, the definitive diagnosis of typhoid fever depends on the isolation of *Salmonella typhi* from blood, stool, urine, and other body fluids [1]. Previous studies show that the prevalence of typhoid fever in 2003 was 2.5% while recent data on the prevalence of typhoid fever is still unavailable. To address this gap of information, a cross-sectional study at the Saint Elisabeth General Hospital Shisong, Bui Division, North West region of Cameroon was carried out to assess the prevalence of typhoid and paratyphoid fever among patients and their awareness of the sources of contamination and means of prevention.

## Methods

**Study design:** this was a cross-sectional descriptive study; conducted at the Saint Elisabeth General Hospital Shisong, from March 1^st^, 2017 to May 31^st^, 2017.

**Study area:** the Saint Elizabeth General Hospital Shisong is situated in Kumbo Town, in the Bui Division, of the Northwest region of Cameroon. It is a referral hospital administrated by the Tertiary Sisters of Saint Francis. It has a staff capacity of over 200 health personnel and 14 medical doctors (11 in the General Hospital and 3 in the cardiac center). The hospital provides health care to a floating population of over 200,000 people.

**Study population:** patients who visited the hospital, presenting with clinical signs and symptoms of typhoid fever and from whom a stool culture was requested to be done by the medical doctor throughout the study period.

**Inclusion criteria:** 1) patients visiting the hospital at the time of the data collection, with clinical signs and symptoms of typhoid fever and of whom a stool culture was requested. 2) Patients must have had a fever for at least 7 days before sample collection.

**Exclusion criteria:** 1) patients on antimicrobial therapy were not included. 2) Patients who did not give their consent.

**Sample size calculation:** a minimum sample size of N=38 was calculated using the Lorentz formula [8] and the Nsutebu *et al*. estimate (2,5%) of the prevalence (P) of typhoid fever in Cameroon [9]. Normal distribution was anticipated with a 95%, confidence interval of statistical significance (a=0.05) and the degree (D) of precision level set to 0.05 for a 95% confidence interval. The sample size of the study was 172 patients, making it the desired sample size.

**Data/sample collection:** a questionnaire designed to capture information on the Knowledge/awareness, attitude, and practices of typhoid fever was administered to all consenting participants. About 1g of stool samples was collected from the same patient in a labeled universal (plastic) disposable tube with a screw cap and transported for culture.

**Stool culture:** Gram staining was performed on all the stool samples to estimate the flora of which if we have 70% bg- and 30% of other bacteria, we will not proceed with culture (normal flora). But if we have 70% bg+ and 30% others, we proceeded with the culture by inoculating stool samples unto MacConkey agar and SS agar. The culture plates were incubated at 37°C for 24 h. The absence of any lactose negative organism after 24 h was labeled negative and if colonies of lactose negative organisms which showed pale color on MacConkey are seen, then colonies with black centers were checked on *Salmonella*-*Shigella* agar (*Salmonella* shows black on all the colonies which indicate the presence of hydrogen sulphide (H2S)). Swarming was also checked on the media to exclude Proteus. Colonies suspected to be *Salmonella* were sub-cultured unto KIA and incubated for 24h at 37°C. *Salmonella* shows a pink slope and a yellow butt with cracks and blackening in the KIA media. Colonies suspected to be *Salmonella* were Gram stained, motility test, Indole test, and Oxidase test performed on the isolates

**Statistical analyses:** the data obtained were entered in duplicates and analysed using Epi info version 7.1.3.3 and Microsoft excel 2010. Correlation between variables was examined using Pearson´s product-moment correlation. The proportion of patients diagnosed with *Salmonella typhi* and with adequate knowledge on means of contamination of *Salmonella typhi* was estimated at a 95% CI. Participants´ knowledge and awareness of typhoid fever were grouped into two categories based on their knowledge and awareness score. Participants whose scores were equal to or greater than the mean score were considered to have correct knowledge and awareness those whose scores were below the mean score were considered to have incorrect knowledge/awareness on typhoid fever.

**Ethical consideration:** ethical clearance was sought and obtained from the National Ethical Committee for Research on Human Health under the University of Dschang, Cameroon with reference number 1807|17|UDs|FS|D|SBM. The administrative authorisation was obtained from the Hospital administration of the Saint Elizabeth General Hospital Shisong. Issues about the autonomy, protection, and confidentiality of the subjects were fully respected.

## Results

**Socio-demographic characteristics of the study population:** most of the participants (164, 94.8%) were adults, 20 years or older a majority (102, 70%) of which were men. A majority of the participants (102, 59.3%) came from the North West region followed by the West region (22, 12.8%). Fifty-seven (33.0%) of the participants had attained secondary education, 46 (26.0%) primary education, and 25 (14.5%) higher education (Table 1).

**Table 1:** socio-demographic characteristics of the study population

Serial number	Socio-demographic characteristics	Number	Percentage (%)
**Level of Education**			
1	Higher education	25	14.5
2	Secondary education	57	33.0
3	Primary education	45	26.0
4	No formal education	46	26.5
**Region**			
1	Center	8	4.7
2	Littoral	15	8.7
3	North	6	3.5
4	North west	102	59.3
5	South west	19	11.1
6	West	22	12.8
**Sex**			
1	Male	121	70.0
2	Female	52	30.0
**Age**			
1	0-9 years	4	2.3
2	10-19 years	5	2.9
3	20+	164	94.8
**Marital status**			
1	Married	110	63.6
2	Single	63	36.4

**Prevalence of typhoid fever:** out of the 173 patients that participated in the study, 52 (30.2%) showed positive culture with the most prevalent species being Salmonella (49, 94.2%) and the least being Salmonella paratyphoid A (3, 5.8%). The Northwest region of Cameroon showed the highest prevalence of patients diagnosed with positive culture (30.4%), followed by the patients from the west region (9 out of 22, 40.9%) (Table 2). There was no significant difference between patients with no education and patients with primary education or above regarding the prevalence of positive culture (6, 24.0% and 17, 37.0% respectively) (Table 3). It was also noted that the majority of patients with a positive stool culture were males (Table 4). The proportion of male patients with positive culture was 31 (59.6%) and 21 (40.4%) for female patients.

**Table 2:** prevalence of patients with positive culture by region of origin

Serial number	Region of origin (N)	Frequency + stool culture (%)	95% LCI	95% UCI
1	Center(8)	2(25.0)	3.2	65.1
2	Littoral(15)	2(13.3)	1.7	40.5
3	North(6)	2(33.3)	4.3	77.7
4	Northwest(102)	31(30.4)	21.7	40.3
5	Southwest (19)	6 (31.6)	12.6	56.6
6	West(22)	9(40.9)	20.7	63.7

**Table 3:** prevalence of patients with positive culture by the level of education

Serial number	Level of education(N)	Frequency +(%)	95% LCI	95% UCI
**1**	Higher education (26)	6(24.0)	9.4	45.1
**2**	Secondary education (57)	14 (24.6)	14.1	37.8
**3**	Primary education (45)	15(33.3)	20.0	49.0
**4**	No formal education (46)	17 (37.0)	23.2	52.5

**Table 4:** prevalence of patients with positive culture by sex

Serial number	Sex (N)	Frequency + stool culture (%)	95% LCI	95% UCI
**1**	Male	31(59.6)	45.1	73.0
**2**	Female	21(40.4)	27.0	54.9

**Awareness of patients on typhoid fever:** while typhoid fever remains a serious problem in sub-Saharan Africa many people from our study remain uninformed about the disease and this unawareness is greatly correlated with the level of education of patients. Patients with no formal education were shown to have less information on typhoid fever (27, 58.7%) compared to those with a higher level of education (25, 100.0%) (Table 5).

**Table 5:** patients’ awareness of typhoid fever and the means of contamination of the disease

Serial number	Education level (N)	Awareness of typhoid fever (%)	Means of contamination	
			Contaminated food (%)	Contaminated water (%)
**1**	Higher education (26)	25 (100.0)	23(92.0)	23(92.0)
**2**	Secondary education (57)	54(94.7)	44(77.2)	41(71.9)
**3**	Primary education (45)	39 (86.7)Table	26(57.8)	21(46.7)
**4**	No formal education (46)	27(58.7)	12(26.1)	11(23.9)

**Knowledge of patients on modes of transmission of typhoid fever:** awareness of typhoid fever being contracted through the consumption of contaminated water and food was shown to be positively correlated with the level of education among patients (Table 4). Twenty-three (92.0%) of the patients who had attained a higher level of education responded that typhoid fever can be contracted through contaminated water, followed by 21 (46.7%) for those who had attained primary education and 11 (23.9%) for patients with no formal education.

## Discussion

The prevalence of typhoid fever was very high at 52 (30.1%) with males being the most affected. The level of education turns out to have a high impact on the mastery of the disease but fairly affected the rate at which the disease is contracted. Social circumstances facilitate the transmission of typhoid fever, and so, individuals in endemic areas are at risk of contracting the disease. Acute infection surpasses chronic ones [10, 11]. In this study, the prevalence of typhoid fever determined using stool culture was 30.1% (CI 23.3-37.5%), this contradicts the report of Nsutebu *et al*. [9] that reported the prevalence of typhoid fever at 2.5% in Cameroon. Our results were similar to a study carried out by Ammah *et al*. [12] who reported the prevalence of typhoid fever at 26%. These findings conclude that Kumbo West and Kumbo East health districts are endemic zones for typhoid fever. Typhoid fever; a potentially life-threatening illness caused by *Salmonella typhi* bacterium necessitates a sustainable intervention to minimize the risk. Nevertheless, complications may occur even if the patients received adequate treatment. Hence, monitoring for the complications is essential especially for travelers to endemic areas [11].

Risk factors for this disease can either be within the household (typhoid) or outside the household (paratyphoid). Previous studies however have demonstrated an association between poor hygiene and carriers of *Salmonella typhi* in families with children who had typhoid fever. In an Indonesian study, poor hygiene was found to be a risk factor for typhoid fever [13]: washing hands before eating was an important preventive measure. In other Indonesian studies, lack of soap for hand washing was independently associated with typhoid fever [14]. Crowded household conditions also increased the risk [15]. Typhoid fever remains an important public health problem in Cameroon. Therefore understanding the risk factors of this illness is primordial in the management of this disease [16]. Some of the risks factors identified by this study included a low level of education, poor hand hygiene, and poor knowledge about the disease, especially among the less educated patients. Corrective measures include increasing the awareness of Salmonella infections through education with an emphasis on the modes of contraction, preventive measures as well as water sanitation.

Analysis by sex showed that the proportion of males with positive culture was far higher than that of females. This result is in line with the study carried out by Smith *et al*. [7] in Nigeria but contrary to what was observed by Mohammad *et al*. [6] in Dhaka and Ramyil *et al*. [17], in Jos Metropolis, Plateau State, Nigeria who found that more females were affected than the males. *Salmonella* in the stool occurs only when one becomes a potential carrier of the infection as explained by Ramyil *et al*. [17]. Amongst the *Salmonella paratyphi, Salmonella paratyphi* A, was the most common species which is in line with the findings of Agbulu *et al*. [18] who argued that this microorganism has been reported to cause typhoid fever in some areas in Asia, China, and India.

**Limitation:** this study cannot be generalized to the population of the Northwest region of Cameroon as it recruited participants from one health facility. More so, the data obtained were driven by the decision of a medical doctor to take stool cultures. Some cases were lost due to not obtaining stool cultures either because the patients refused to do the test or did not have money to carry out the test. The Saint Elisabeth general hospital Shisong is a reference hospital and the majority of the poor people in the community will prefer smaller clinics where consultation fees and prices for medications are a bit lower.

**Recommendations:** the health systems in Cameroon, as in most endemic areas, need to be strengthened to be able to deliver appropriate services as a public health preventive measure, intensive community health education needs to be integrated into typhoid fever disease control protocol.

## Conclusion

A high prevalence of typhoid fever was observed in our study. The unawareness of the patients on typhoid fever and its contraction through contaminated water and food was positively correlated to the level of educations of the patients. These findings, therefore, suggest a public health challenge faced by inhabitants in this region where typhoid fever remains endemic. Scarcity of potable water, improper drainage systems, and problems of unsanitary toilets in Cameroon require urgent intervention.

### What is known about this topic

- The prevalence of typhoid fever is aggravated by rapid population growth;
- Stool excretion of Salmonella typhi during and after infection is the main source of the infection;
- Typhoid fever occurs in all age groups.

### What this study adds

- This study will bring out the most recent prevalence of typhoid fever in Cameroon;
- This study will help policymakers to have a new look at typhoid fever as previous studies have shown that Cameroon is not an endemic zone;
- Findings from this study would urge researchers to carry out more research on this domain.
